# Pulse energy measurement at the SXR instrument

**DOI:** 10.1107/S1600577515006098

**Published:** 2015-04-14

**Authors:** Stefan Moeller, Garth Brown, Georgi Dakovski, Bruce Hill, Michael Holmes, Jennifer Loos, Ricardo Maida, Ernesto Paiser, William Schlotter, Joshua J. Turner, Alex Wallace, Ulf Jastrow, Svea Kreis, Andrey A. Sorokin, Kai Tiedtke

**Affiliations:** a Linac Coherent Light Source, SLAC National Accelerator Laboratory, 2575 Sand Hill Road, Menlo Park, CA 94025, USA; b Deutsches Elektronen-Synchrotron, DESY, Notkestrasse 85, D-22603 Hamburg, Germany; c Ioffe Physico-Technical Institute, Polytekhnicheskaya 26, 194021 St Petersburg, Russia

**Keywords:** FEL, diagnostics, pulse energy detector

## Abstract

A gas monitor detector was implemented and characterized at the Soft X-ray Research (SXR) instrument to measure the average, absolute and pulse-resolved photon flux of the LCLS beam in the energy range between 280 and 2000 eV. The detector is placed after the monochromator and addresses the need to provide reliable absolute pulse energy as well as pulse-resolved measurements for the various experiments at this instrument. This detector provides a reliable non-invasive measurement for determining flux levels on the samples in the downstream experimental chamber and for optimizing signal levels of secondary detectors and for the essential need of data normalization. The design, integration into the instrument and operation are described, and examples of its performance are given.

## Abstract 2

A gas monitor detector was implemented and characterized at the Soft X-ray Research instrument (SXR) to measure the average, absolute and pulse-resolved photon flux of the LCLS beam in the energy range between 280 and 2000 eV.

## 1. Introduction   

The advancement of beam diagnostics needs to keep pace with the rapid development of X-ray free-electron laser (FEL) sources in the recent and coming years. Absolute photon flux is one important parameter for scientific experiments especially for SASE (self-amplified spontaneous emission)-generated FEL pulses which are of chaotic nature and thus require a real-time pulse-resolved diagnostic. Established methods such as calorimetry intercept the beam and do not provide the pulse resolution (Kato *et al.*, 2009 ▶). Furthermore, the intense and short-pulse FEL beam can damage materials or at least degrade their properties. The gas monitor detector (GMD) used here is gas-based and fulfills these requirements. It is non-invasive, measures single pulses, and provides a wide dynamic range from spontaneous to saturated SASE levels. It was developed and tested by a group at the Deutsches Elektronen-Synchrotron (DESY), Physikalisch-Technische Bundesanstalt (PTB) and Ioffe Institute (Richter *et al.*, 2003 ▶; Tiedtke *et al.*, 2008 ▶). A prototype modified for the soft X-ray range has been tested and characterized at the LCLS previously (Tiedtke *et al.*, 2014 ▶). A permanent device has recently been integrated into the instrument and commissioned. The detector needs to be reliable and adaptable to widely different operational conditions: such as, for example, pink (zero-order mode) or monochromatic beam (first-order mode), including high-resolution settings covering the photon energies 280–2000 eV. Other examples include short-pulse machine mode resulting in over ten times lower pulse energies, and FEL operation with seeded beam. The detector supports operation of machine scanning of the FEL photon energies which is essential for spectroscopy methods as well as responding adequately to changing pulse rates from 1 to 120 Hz which will affect measurements of the average pulse energy.

In this article we review the operational principle of the detector and describe its design and integration into the Soft X-ray Research (SXR) instrument. We describe signal processing and operational features for supporting user experiments. Finally, we show some examples of measurements demonstrating its performance.

## 2. Detector description   

**Figure 1:**
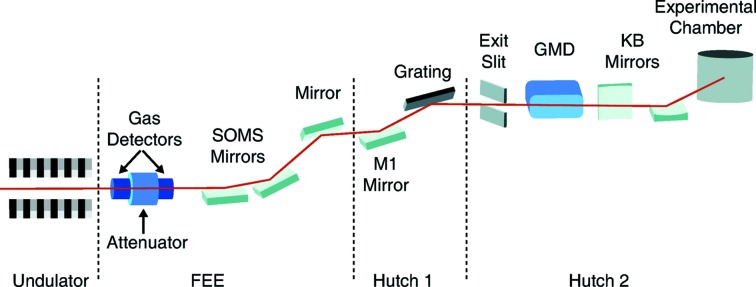
Optical lay-out of the SXR instrument. The front-end enclosure (FEE) includes the two LCLS gas detectors with attenuator system and the offset and steering mirrors. The SXR monochromator is located in hutch 1 (AMO hutch) with its exit slit in hutch 2 (SXR hutch) which is followed by the GMD, the KB mirrors and experimental chamber.

The lay-out of the SXR instrument is schematically shown in Fig. 1 ▶. The FEL beam exits the LCLS undulators and traverses the front-end enclosure (FEE) which houses, among other diagnostics, the LCLS gas attenuator and integrated gas detectors (Moeller *et al.*, 2011 ▶). Several offset and steering mirrors guide the beam through the first hutch which houses the SXR monochromator (Heimann *et al.*, 2011 ▶). The exit slit of the monochromator is located in the SXR hutch followed by the SXR GMD detector. A Kirkpatrick–Baez (KB) focusing optics separates it from the experimental chamber.

The SXR GMD is placed after the monochromator. In zero-order mode of the monochromator, a good correlation with the FEE gas detectors is expected (see §3). However, in first-order mode the beam intensity does not necessarily correlate any longer with the incoming beam intensity depending on the bandwidth setting of the exit slit as each SASE pulse jitters in pulse and photon energy. Beam loss on the mirrors and apertures further necessitates an intensity detector directly at the SXR instrument in addition to the existing energy detectors in the FEE which are calibrated with a so-called electron beam loss method (Moeller *et al.*, 2011 ▶).

Knowing the photon flux directly at the sample location is of greatest interest. However, due to the constraints of the KB mirror system, the GMD could not be moved closer to the sample chamber. Placing the detector downstream was also not an option as the SXR chamber uses mainly solid samples. In order to account for the KB optics, we have separately measured the transmission of the KB mirrors to be close to 50% over the soft X-ray energy range.

### 2.1. Gas monitor detector principle   

**Figure 2:**
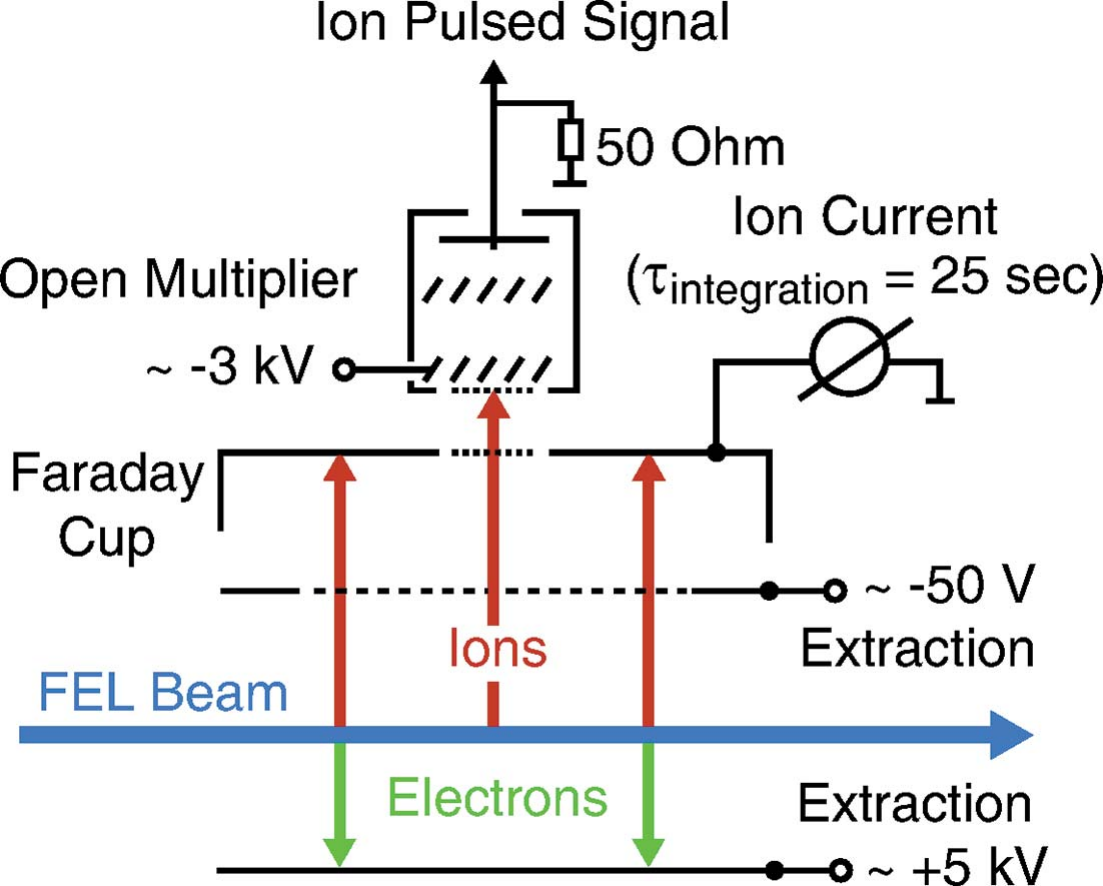
Schematic diagram of the gas monitor detection. The blue arrow represents the FEL beam. The red arrows represent the ion and the green arrows the electron trajectories. The applied high-voltage scheme and the signal readout channels are shown. Figure reprinted with permission from Tiedtke *et al.* (2014 ▶).

The detector principle is based on photoionization of rare gas atoms and the detection of the created ions. The operation principle of the detector is described by Tiedtke *et al.* (2014 ▶) and depicted in Fig. 2 ▶.

**Figure 3:**
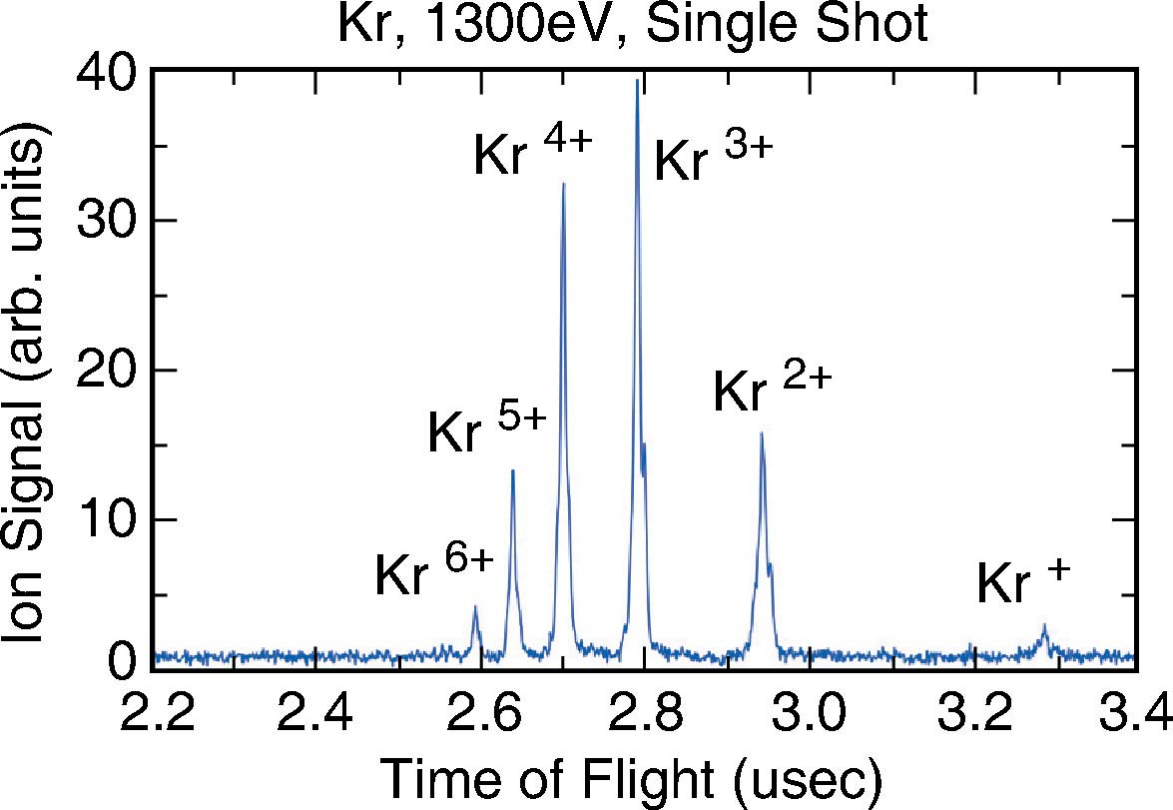
Single-pulse ion time-of-flight spectrum (uncorrected) for krypton at 1300 eV.

Following photoionization with the FEL pulse of the target gas (at low pressures from 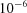 to 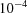
 mbar) the created ions and electrons are separated and fully extracted by applied homogeneous static electric fields. The ions are collected by a simple cup-shaped metal electrode (Faraday cup) ensuring linear response over a wide dynamic range, and the current is measured *via* an adapted RC filter by a calibrated pico ampere meter (Keithley 6514). The Faraday ion current provides a time-averaged (time constant 25 s) calibrated signal corresponding to the number of ions collected by the electrode. This relation has been externally calibrated against reference standards (Beckhoff *et al.*, 2009 ▶). A slit in the Faraday cup transmits a fraction of the ions which are measured pulse-resolved with an open electron multiplier (type ETP 14880 from SGE) with an amplification factor of up to 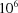. This secondary detection channel using a multiplier provides the required sensitivity allowing operation in the soft X-ray regime. The integrated waveform of the multiplier signal provides a relative pulse-resolved signal which can be correlated against the absolute average ion current signal. The multiplier can also be operated in time-of-flight (TOF) mode to resolve different ion charge states. Averaged spectra and correcting for the detection efficiency due to different ion charge states allows for deducing mean charge values when not available in the literature. Mean charge values are tabulated for portions of the soft X-ray region (Suzuki & Saito, 1992 ▶). An example of a single-pulse TOF spectrum is shown in Fig. 3 ▶.

The number of photons passing through the detector can be expressed as (Richter *et al.*, 2003 ▶)
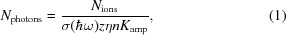
where 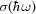 is the total photoionization cross section [tabulated values given by Tiedtke *et al.* (2008 ▶); Suzuki & Saito (2002 ▶, 2003 ▶, 2005 ▶); Saito & Suzuki (2001 ▶); Henke *et al.* (1993 ▶)], *z* is the effective detection length, η the ion detection efficiency, *n* is the gas density which is measured according to the relation *n* = 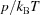, where *p* is the pressure [measured with a precision spinning rotor gauge (SRG-3 from MKS) and calibrated to include adjustment factors for the rare gases], *T* is the temperature (measured with a Pt100 resistance thermometer), and 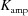 is the detector’s amplification factor (unity for the Faraday cup). The number of ions 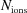 is related to the measured charge *Q* by normalizing by the mean charge state of the detected ions. The remaining unknown parameters *z* and η are predetermined by measurement against an external calibration standard performed at the Radiometry Laboratory of the PTB at the electron storage ring BESSY in Berlin using monochromated synchrotron radiation and a calibrated semiconductor photodiode as the secondary standard (Richter *et al.*, 2003 ▶; Tiedtke *et al.*, 2008 ▶).

### 2.2. Mechanical   

**Figure 4:**
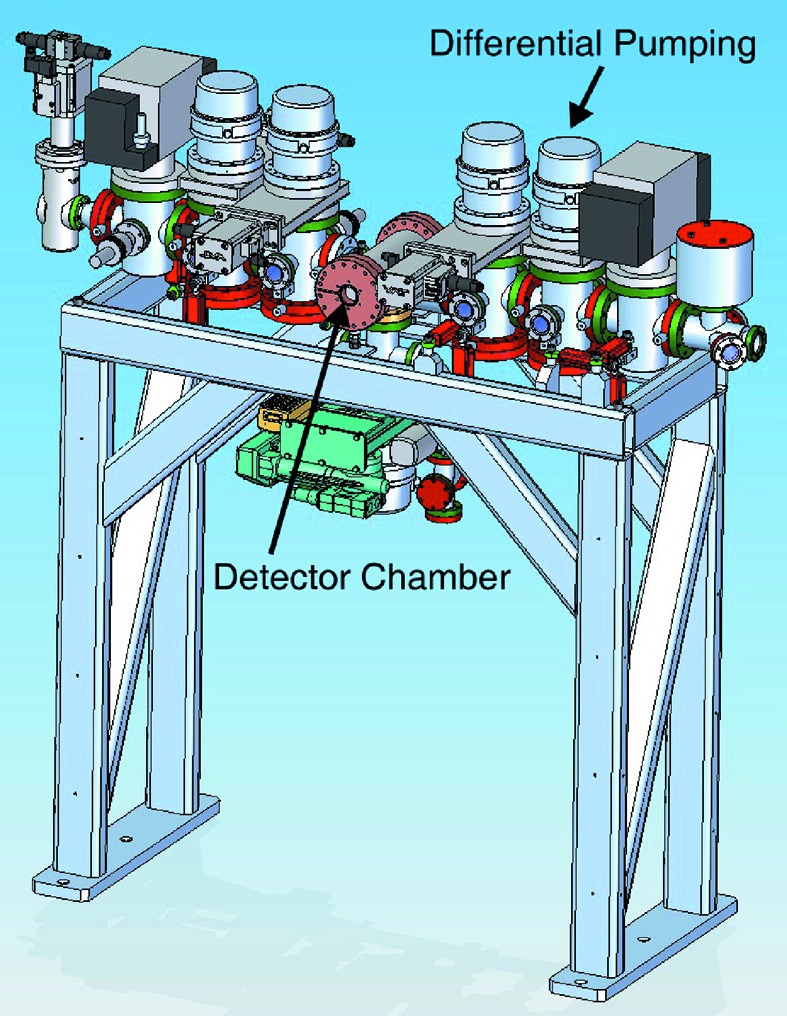
Schematic of the mechanical assembly. The detector is located in the center chamber with differential pumping stages on the upstream and downstream side. The upstream aperture assembly and downstream gate valve with stand and strut system are also shown.

The entire SXR GMD detector assembly is about 160 cm in length and consists of the main detector chamber (center chamber in Fig. 4 ▶) and the differential pumping system. The detector chamber contains the detectors (Faraday cup and multiplier), high-voltage meshes and the spinning rotor gauge. The three-stage differential pumping system isolates the gas volume in the detector chamber from the instrument vacuum on either side. A total of eight differential pumping tubes with an oval inner diameter of 10.3 mm in height and 7.7 mm in width separate the pumping stages. An upstream aperture of equal dimensions consisting of a YAG crystal backed by 10 mm-thick boron carbide provides burn through protection from the FEL beam to the stainless steel tubes. The oval shape takes into account the vertical beam movement possible by the monochromator optics. The tubes can be aligned individually during assembly. A strut system allows for aligning the chamber assembly with respect to the frame and locks it into position.

### 2.3. Vacuum and controls   

Upstream and downstream gate valves separate the unit from neighboring devices. The main chamber is pumped with a turbo pump (Varian 250 L s^−1^) through a variable gate valve. The two neighboring zones are also pumped with turbo pumps which can be isolated by gate valves while the outer zones are pumped with ion pumps (Vacion Plus 75L). With an operating pressure of 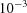
 mbar in the main chamber, the pressure in the outer zone remains in the low 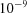
 mbar range. Routine operating pressure is in the low 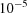
 mbar range. All zones are equipped with Pirani and cold cathode gauges with a subset of them included in the vacuum interlock system.

The GMD vacuum and gas controls use a combination of PLC (Programmable Logic Control) and EPICS (Experimental Physics and Industrial Control System). A Beckhoff PLC is used to enforce interlocks and implement a purge/switch gas manifold routine. EPICS is used to provide archiving of the instrument state and the remote operator interface.

#### 2.3.1. Gas manifold and inlet system   

The gas inlet system allows the user to select from four different gases at the push of a button. A dosing valve (Pfeiffer EVR 116) is used to control the gas flow from the gas manifold supply. The dosing valve operates in open loop mode for better stability as the supply is functionally a constant pressure source. A proportional gate valve (VAT) actuated by a stepper motor allows gas flow to be reduced into the main chamber for extended supply lifetime and pressure stabilization.

#### 2.3.2. Purge cycle and changing gases   

Selecting a gas in the EPICS user panel initiates an automatic routine that closes the current supply valve (if switching gases), purges the gas manifold, pumps trapped gas volumes in the needle dosing valve and opens the selected gas supply solenoid. To avoid cross contamination it is important to clear the gas supply manifold and dosing valve of other gases. At one point in the automated routine, the dosing valve is opened to 100% and pumped by the interaction chamber for roughly a minute.

#### 2.3.3. Interlocks   

The interlocks serve to protect components upstream and downstream of the vacuum system, and the pumps of the GMD itself, prioritized in that order. Additionally, the system is robust enough to survive un­intentional pressure spikes and maintain overall instrument operation.

The vacuum gauges in each differential section of the GMD have up to four available pressure set points for use. When the interaction chamber pressure rises above a moderate pressure, a safety valve upstream of the dosing valve closes, along with the dosing valve. The differential section turbo pumps and upstream/downstream instrument valves remain open. This allows the GMD to continue operation and potentially recover. If the pressure rises even higher and breaches the secondary set-point, the interaction chamber turbo is isolated from the volume and the upstream/downstream isolation valves are closed. At this point each differential pumping section turbo can remain online until its individual secondary set-point is breached.

### 2.4. Signal processing   

The TOF multiplier waveforms are digitized (Acqiris U1065A from Agilent) on a pulse-by-pulse basis (120 Hz maximum LCLS repetition rate). An example of a single-shot waveform is shown in Fig. 3 ▶ using krypton as the target gas at 1300 eV in the zero-order monochromator setting. The different charge states are clearly visible. On each pulse the individual peaks of the spectrum are integrated, and a noise background correction is applied choosing a signal-free section of the waveform prior to the incoming signal. The integration limits are user defined in a graphics panel, and presets can be applied depending on the target gas and multiplier voltage. For each target, gas presets are available that apply settings optimized for various experimental conditions and signal levels. Common modes of operation are: zero- and first-order monochromator mode, and first-order high-resolution settings. This can be combined with low-charge and short-pulse LCLS machine operation. Individual ion charge peaks can be selected to be included in the integrated signal, in order to account for the dependence of the charge distribution on photon energy. All settings are also configurable *ad hoc* to optimize for special beam conditions, and new settings can be saved and reapplied.

A number of parameters such as integrated waveform signal, background and background-corrected signal are calculated and recorded on a fast data stream per pulse basis for data post-processing. Optionally, the raw waveforms can be recorded for each shot for users to apply specialized calibration techniques. Pulses can be correlated to the average current signal to provide a pulse calibration (in mJ) by applying a filter on the pulses due to the slower Keithley picometer and RC unit response.

In addition, LCLS machine parameters are logged as process variables such as photon energy, pulse rate, pulse length and the FEE gas detector signals, in order to be able to correlate with the GMD signal that may affect scientific data. Slow varying parameters such as target gas pressure and temperature are also recorded. For calculating the number of photons per pulse, a look-up table provides the cross section and mean charge values for a given energy. When operating near absorption edges of the target gas, an alert message prompts the user to change gases.

## 3. Performance   

**Figure 5:**
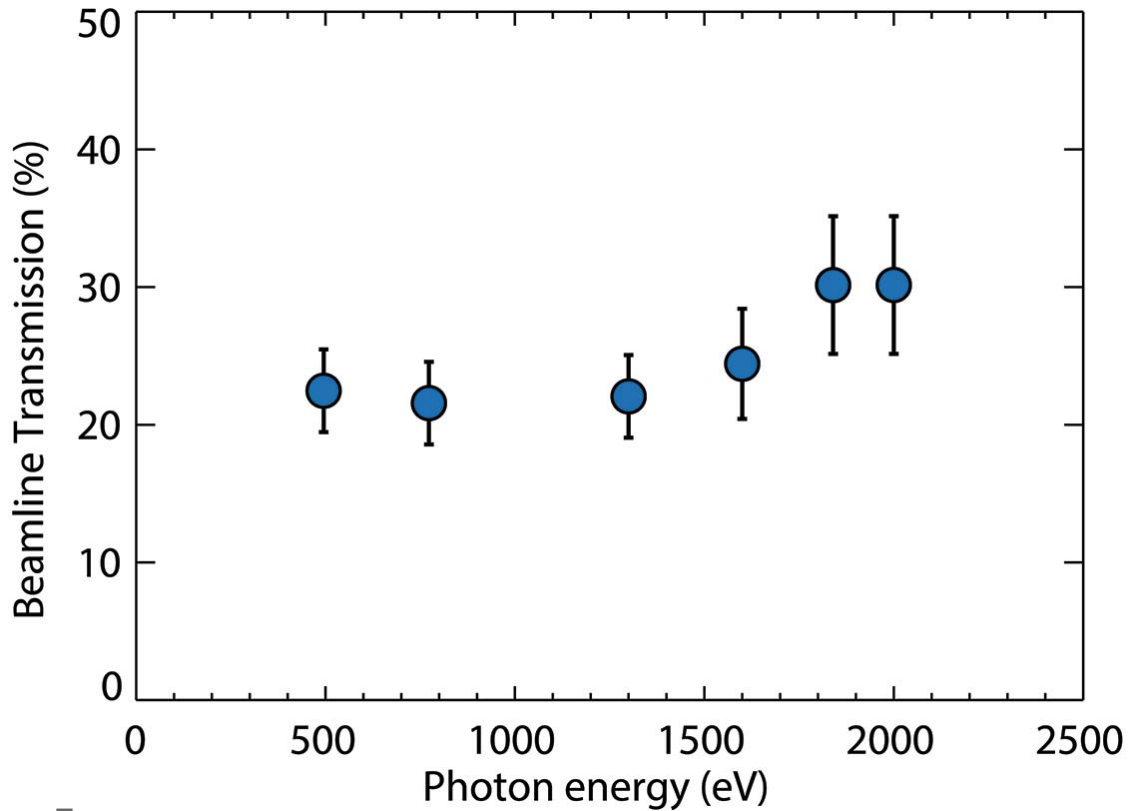
Measured transmission of the SXR instrument in zeroth order of the monochromator (transmission of the KB optics is not included). The error bars combine the variability of the limited measurement points and the estimated 5% uncertainty of the pulse energy measurement.

One of the fundamental quantities of an X-ray instrument is the average transmission as a function of photon energy. The measured values in Fig. 5 ▶ for photon energies from 500 to 2000 eV show a comparison between the average ion current signal of the SXR GMD and the upstream FEE gas detector. Measurements were obtained in the zero order of the monochromator and with fully open exit slits for various attenuator settings of the gas attenuator. Values do not include the transmission of the KB optics. The transmission is between 20 and 30% over the entire range of photon energies. The transmission values from Fig. 5 ▶ are listed again for reference in Table 1 ▶ in addition to the average number of photons per pulse per millijoules.

Earlier instrument commissioning results had shown similar values; however, those included the transmission of the KB optics (Tiedtke *et al.*, 2014 ▶). Beam clipping on mirrors and apertures had been attributed to this behavior. Therefore we expect that, over the five years of operation, the optics surfaces have further degraded (due to carbon deposition which in fact can be observed on the mirror surfaces) and lowered the transmission further. With perfect surfaces about 45% transmission is expected, assuming that the five instrument optical elements upstream of the SXR GMD (Fig. 1 ▶) have an average reflectivity of 85% (Soufli *et al.*, 2012 ▶).

The increasing transmission towards higher energies is surprising given that transmission calculations for the five upstream B4C-coated silicon mirrors show decreasing transmission towards 500 and 2000 eV similiar to earlier results (Tiedtke *et al.*, 2014 ▶). Adding a carbon contamination layer to simulations does not significantly alter this behavior and thus cannot account for our measured transmission curve. The transmission curve may suggest that beam clipping on the upstream mirrors could play a role. As the beam size decreases with increasing photon energy the clipping would be less noticeable here resulting in a higher transmission. The FEE gas detectors are located upstream of the beam optics and would therefore be unaffected. However, this clipping effect implies that the beam trajectory was markedly different in comparison with other beam conditions of earlier results. Typically only very few experiments at SXR use the higher photon energies close to 2000 eV. Nevertheless, we plan to study this effect more systematically in the future.

Commissioning of the SXR GMD detector included a series of measurements that first demonstrated baseline performance. For example, all four rare gases were used for selected photon energies and similar conditions to demonstrate comparable detector output. The photoionization cross sections of the gases differ by up to an order of magnitude in this photon energy range; however, the reproducibility of transmission values had an error of less than 5%. An error estimate of the relative standard uncertainty of the measured pulse energy gives about 6%. This includes uncertainties of the separate pressure and temperature measurements, the effective length, mean charge values and cross-section values. Thus the transmission values have an uncertainty of less than 10%. Note that the results in Table 1 ▶ and Fig. 5 ▶ also include the uncertainty of the FEE gas detector.

The detection method of this version of the SXR GMD has been validated in several studies and compared with calorimetric techniques very successfully demonstrating that the synchrotron-based calibration of the average ion current signal is valid (see, for example, Saito *et al.*, 2010 ▶; Kato *et al.*, 2012 ▶). Furthermore this was also shown for previous measurements with the prototype GMD at SXR comparing it with the FEE gas detectors. More recent tests demonstrated that the average ion current signal has a sensitivity better than 0.1 µJ.

The TOF ion pulse-resolved measurements are crucial for normalizing downstream experiment detectors with the incoming intensity. As shown in Fig. 3 ▶, the strong and well resolved single-pulse ion TOF signals allow the implementation of a robust calibration method to optimize the signal-to-noise ratio. This has made it easier to perform routine calibration before and during each experiment.

**Figure 6:**
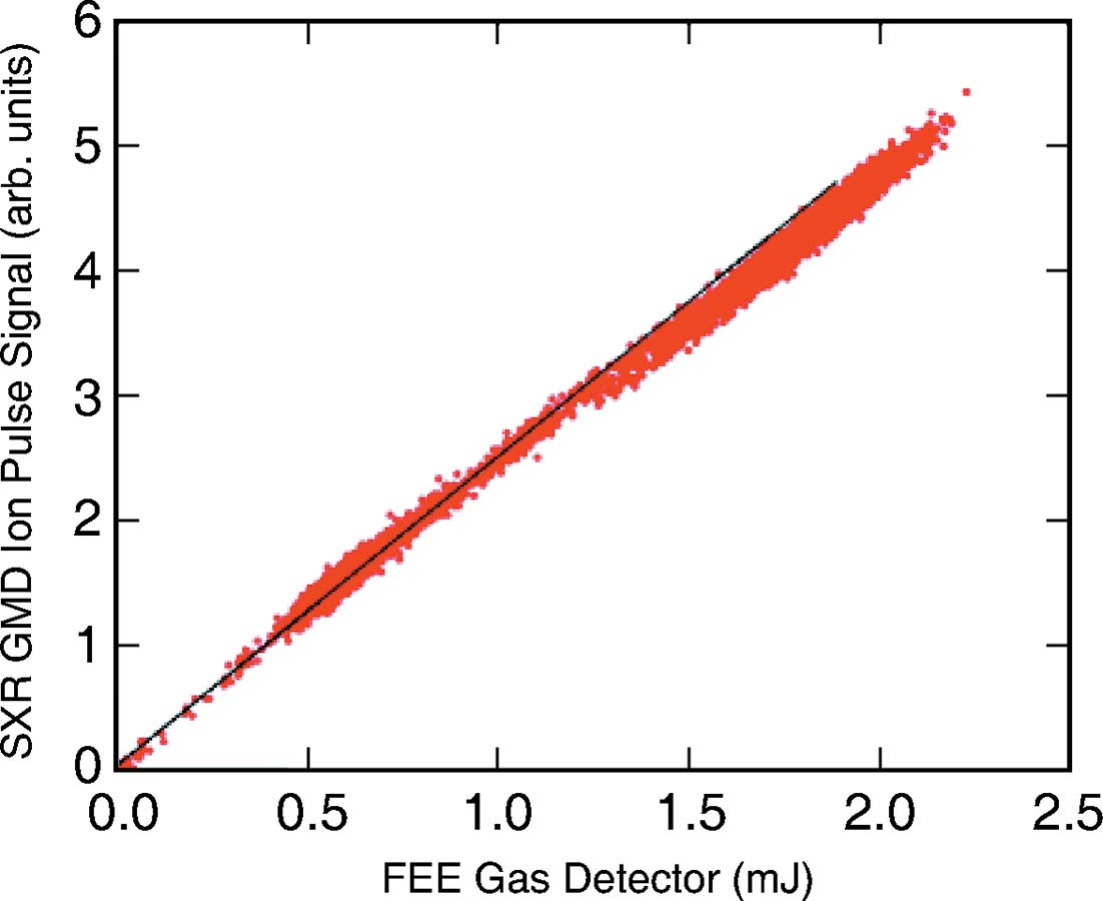
Ion pulse signal *versus* FEE gas detector correlation for varying attenuation levels. Target gas argon, photon energy 1300 eV, zero-order monochromator mode. A linear fit (black straight line) shows the deviation at higher pulse energies.

In Fig. 6 ▶ we show a correlation of the TOF pulse signal with the upstream FEE gas detector in zeroth order of the monochromator. The data span a wide range over which the gas attenuator settings were changed continuously. The overall behavior is quite linear. As can be seen, a linear fit (black straight line) shows a deviation at higher pulse energies (greater than about 1.5 mJ of incoming intensity). Separate plots of the GMD signal and the FEE gas detector signal *versus* the gas attenuator pressure settings show perfect linearity in the case of the GMD and a slight non-linearity for the FEE gas detector correlation indicating that the small deviation stems from the FEE gas detector.

In the near future we plan to implement a comprehensive pulse-by-pulse calibration based on the average ion current signal which will need to span a wide range of operating conditions with respect to the beamline as well as the LCLS machine settings.

## 4. Conclusion   

We described the design, integration and performance of the SXR GMD at the Soft X-ray Research instrument at LCLS. The detector, developed by a group from DESY, PTB and Ioffe Institute, has been integrated into SXR where it has become a permanent, integral and indispensable diagnostic tool for performing advanced and complex FEL experiments. We have built a robust vacuum and gas delivery system to ensure reliable operation, and developed controls and signal analysis features that allow flexible user operation adaptable to a wide range of beam conditions.

## Figures and Tables

**Table 1:** Measured transmission and average number of photons per pulse per mJ of the SXR instrument in zero-order mode of the monochromator (KB optics not included)

Photon energy (eV)	Transmission (%)	Average number of photons per pulse per mJ
495	23 3	2.8 0.4 10^12^
773	22 3	1.7 0.3 10^12^
1300	22 3	1.1 0.2 10^12^
1600	24 4	1 0.1 10^12^
1840	30 5	1 0.2 10^12^
2000	30 5	0.9 0.1 10^12^

## References

[bb1] Beckhoff, B., Gottwald, A., Klein, R., Krumrey, M., Müller, R., Richter, M., Scholze, F., Thornagel, R. & Ulm, G. (2009). *Phys. Status Solidi B*, **246**, 1415–1434.

[bb2] Heimann, P., Krupin, O., Schlotter, W. F., Turner, J. J., Krzywinski, J., Sorgenfrei, F., Messerschmidt, M., Bernstein, D., Chalupský, J., Hájková, V., Hau-Riege, S., Holmes, M., Juha, L., Kelez, N., Lüning, J., Nordlund, D., Fernandez Perea, M., Scherz, A., Soufli, R., Wurth, W. & Rowen, M. (2011). *Rev. Sci. Instrum.* **82**, 093104. 10.1063/1.3633947 21974570

[bb3] Henke, B. L., Gullikson, E. M. & Davis, J. C. (1993). *At. Data Nucl. Data Tables*, **54**, 181–342.

[bb4] Kato, M., Saito, N., Tanaka, T., Morishita, Y., Kimura, H., Ohashi, H., Nagasono, M., Yabashi, M., Tono, K., Togashi, T., Higashiya, A. & Ishikawa, T. (2009). *Nucl. Instrum. Methods Phys. Res. A*, **612**, 209–211.

[bb5] Kato, M., Tanaka, T., Kurosawa, T., Saito, N., Richter, M., Sorokin, A. A., Tiedtke, K., Kudo, T., Tono, K., Yabashi, M. & Ishikawa, T. (2012). *Appl. Phys. Lett.* **101**, 023503.

[bb6] Moeller, S. *et al.* (2011). *Nucl. Instrum. Methods Phys. Res. A*, **635**, S6–S11.

[bb7] Richter, M., Gottwald, A., Kroth, U., Sorokin, A., Bobashev, S., Shmaenok, L., Feldhaus, J., Gerth, C., Steeg, B., Tiedtke, K. & Treusch, R. (2003). *Appl. Phys. Lett.* **83**, 2970.

[bb8] Saito, N., Juranić, P., Kato, M., Richter, M., Sorokin, A., Tiedtke, K., Jastrow, U., Kroth, U., Schoppe, H., Nagasono, M., Yabashi, M., Tono, K., Togashi, T., Kimura, H., Ohashi, H. & Ishikawa, T. (2010). *Metrologia*, **47**, 21–23.

[bb9] Saito, N. & Suzuki, I. (2001). *Nucl. Instrum. Methods Phys. Res. A*, **467**–**468**, 1577–1580.

[bb10] Soufli, R., Fernández- Perea, M., Baker, S. L., Robinson, J. C., Gullikson, E. M., Heimann, P., Yashchuk, V. V., McKinney, W. R., Schlotter, W. F. & Rowen, M. (2012). *App. Opt.* **50**, 2118. 10.1364/AO.51.002118 22534924

[bb11] Suzuki, I. & Saito, N. (1992). *ETL Tech. Rep.* **56**, 688.

[bb12] Suzuki, I. & Saito, N. (2002). *J. Electron Spectrosc. Relat. Phenom.* **123**, 239–245.

[bb13] Suzuki, I. & Saito, N. (2003). *J. Electron Spectrosc. Relat. Phenom.* **129**, 71–79.

[bb14] Suzuki, I. & Saito, N. (2005). *Radiat. Phys. Chem.* **73**, 1–6.

[bb15] Tiedtke, K., Feldhaus, J., Hahn, U., Jastrow, U., Nunez, T., Tschentscher, T., Bobashev, S., Sorokin, A., Hastings, J., Möller, S., Cibik, L., Gottwald, A., Hoehl, A., Kroth, U., Krumrey, M., Schöppe, H., Ulm, G. & Richter, M. (2008). *J. Appl. Phys.* **103**, 094511.

[bb16] Tiedtke, K., Sorokin, A., Jastrow, U., Juranić, P., Kreis, S., Gerken, N., Richter, M., Arp, U., Feng, Y., Nordlund, D., Soufli, R., Fernández-Perea, M., Juha, L., Heimann, P., Nagler, B., Lee, H. J., Mack, S., Cammarata, M., Krupin, O., Messerschmidt, M., Holmes, M., Rowen, M., Schlotter, W., Moeller, S. & Turner, J. J. (2014). *Opt. Express*, **22**, 21214–21226. 10.1364/OE.22.021214 25321502

